# Neuralgic Amyotrophy and Hourglass Nerve Constriction/Nerve Torsion: Two Sides of the Same Coin? A Clinical Review

**DOI:** 10.3390/brainsci14010067

**Published:** 2024-01-10

**Authors:** Giuseppe Granata, Fabiola Tomasello, Maria Ausilia Sciarrone, Vito Stifano, Liverana Lauretti, Marco Luigetti

**Affiliations:** 1 Dipartimento di Neuroscienze, Organi di Senso e Torace, Fondazione Policlinico Universitario Agostino Gemelli IRCCS, 00168 Rome, Italy; giuseppe.granata@policlinicogemelli.it (G.G.); stifano89@gmail.com (V.S.); liverana.lauretti@policlinicogemelli.it (L.L.); 2 Dipartimento di Neuroscienze, Università Cattolica del Sacro Cuore, 00168 Rome, Italy; fabiolatomasello@hotmail.it (F.T.); a.sciarrone97@gmail.com (M.A.S.)

**Keywords:** hourglass-like constriction, nerve torsion, neuralgic amyotrophy, radial nerve palsy, anterior interosseous nerve palsy, posterior interosseous nerve palsy, neurolysis

## Abstract

Neuralgic amyotrophy, also called Parsonage–Turner syndrome, in its classic presentation is a brachial plexopathy or a multifocal neuropathy, involving mainly motor nerves of the upper limb with a monophasic course. Recently, a new radiological entity was described, the hourglass constriction, which is characterized by a very focal constriction of a nerve, or part of it, usually associated with nerve thickening proximally and distally to the constriction. Another condition, which is similar from a radiological point of view to hourglass constriction, is nerve torsion. The pathophysiology of neuralgic amyotrophy, hourglass constriction and nerve torsion is still poorly understood, and a generic role of inflammation is proposed for all these conditions. It is now widely accepted that nerve imaging is necessary in identifying hourglass constrictions/nerve torsion pre-surgically in patients with an acute mononeuropathy/plexopathy. Ultrasound and MRI are useful tools for diagnosis, and they are consistent with intraoperative findings. The prognosis is generally favorable after surgery, with a high rate of good motor recovery.

## 1. Introduction

Neuralgic amyotrophy (NA), also called Parsonage–Turner syndrome, in its classic presentation is a brachial plexopathy or a multifocal neuropathy involving mainly motor nerves of the upper limb with a monophasic course [1,2,3]. However, from the original report of Parsonage and Turner in 1948, different clinical phenotypes were included in the syndrome. Variants include lower limb plexopathies, sensory–motor involvement and mononeuropathies of different nerves, such as the radial, median, anterior and posterior interosseous and phrenic nerves [4]. Autoimmune, genetic, infectious and mechanical processes are thought to be involved, but etiopathogenesis is still incompletely understood [1].

While the originally described presentation was characterized by spontaneous and good recovery, the broadening of the clinical phenotype also included cases with poor prognosis [5].

More recently, a new radiological entity was described, the hourglass constriction. This entity is characterized by a very focal constriction of a nerve, or part of it (one or more fascicles), usually associated with nerve thickening proximally and distally to the constriction, which involves the perineurium and is associated with marked edema, a mononuclear inflammatory infiltration, composed of CD8-positive T lymphocytes [6,7]. Another condition, similar from a radiological point of view to hourglass constriction, is nerve torsion (in this case, the process can also be partial, involving only few fascicles, or complete, involving the entire nerve) [8]. The most common involved nerves are the radial nerve (RN) along with the posterior interosseous nerve (PIN), its main branch, and the median nerve and its main branch, the anterior interosseous nerve (AIN), while the involvement of the musculocutaneous, the suprascapular and the axillary nerves is rare [9,10,11,12,13]. More rarely, other nerves are involved, and cases of nerve root involvement have been sporadically described in the literature. The clinical picture of both these entities, hourglass constriction and nerve torsion, is usually characterized by a sudden mononeuropathy, usually a severe axonotmesis, documented during neurophysiological examination, sometimes preceded by paraesthesias in the territory of the involved nerve (in the case of a mixed nerve) and pain, even if usually not severe [14]. Typically, hourglass constriction and nerve torsion cause a sensory–motor deficit, in the case of mixed nerve involvement, or only paresis/plegia in the case of pure motor nerve involvement. The prognosis of these conditions is often poor in the absence of any surgical treatment [15].

The pathophysiology of NA, hourglass constriction and nerve torsion is still poorly understood, and a generic role of inflammation is proposed for all these conditions [2,16,17,18].

Due to this common generic role of inflammation and based on some evident similarities between these pathologies from a clinical point of view, some authors proposed hourglass constriction and nerve torsion as a subgroup of NA. Some authors hypothesized that in NA, pathological US findings can be grouped into four categories: nerve swelling, swelling with incomplete constriction, swelling with complete constriction and fascicular entwinement. This may represent a continuum of pathologic processes [19]. According to this view, in some cases of NA, after a first phase characterized by nerve inflammation and swelling, in selected anatomical conditions, the process evolves into hourglass constriction or nerve torsion [6].

However, the picture is still foggy, and at least three important open questions remain:

Are NA, hourglass constriction and nerve torsion really a different evolution of the same pathology?
What is the best diagnostic approach for these conditions?
What is the best treatment approach for these conditions?

To try to respond to these apparently simple questions, it is important to highlight a few milestones based on the data already available in literature and on our personal experience.

## 2. Are NA, Hourglass Constriction and Nerve Torsion Really a Different Evolution of the Same Pathology? 

**Figure 1:**
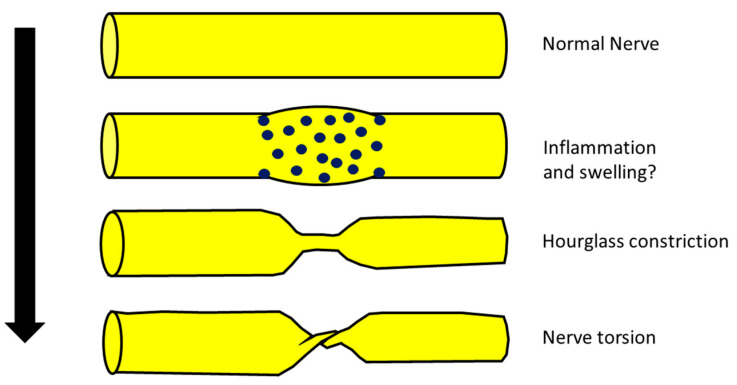
Possible scheme of evolution from a normal nerve, trough inflammation and swelling mediated by mononuclear inflammatory infiltration (blue dots) to hourglass constriction and nerve torsion.

Some authors recently hypothesized that some cases of NA, after a first phase characterized by nerve inflammation and swelling, in particular anatomical conditions, could evolve into hourglass constriction and/or in nerve torsion [8,20] (Figure 1).

However, evidence of this evolution is still lacking since there are no documented cases that have progressed from swelling into hourglass constriction. In other words, there have been no cases diagnosed as NA with initial evidence of nerve swelling at neuroimaging evolving into hourglass constriction or nerve torsion at the same site where previously there was only the swelling. This would be final and decisive proof of the model proposed before. Hence, the hypothesis that hourglass constriction is a particular subtype of NA, even if interesting, is still only a suggestion. Moreover, even if the clinical picture of hourglass constriction/nerve torsion and the classic presentation of NA are similar, some evident differences exist, as shown in Table 1.

(a) Typical NA is characterized by brachial plexus involvement, while hourglass constriction and nerve torsion are mainly found in the radial nerve, PIN and AIN [21,22,23]. Hence, the topography of typical involvement is clearly different, as follows:
(b) The classic clinical phenotype of NA is characterized by strong and invalidating pain that, on the other hand, is not the main feature of hourglass constriction (some pain or sensory symptoms are usually present but not invalidating) [2,24].
(c) From a neurophysiological point of view, the typical picture of NA is the main or exclusive involvement of motor fibers, even in the case of mixed nerve involvement. On the other hand, hourglass constriction/nerve torsion usually affects both motor and sensory fibers at the same time and with the same severity. Motor fibers are exclusively included only in the case of pure motor nerve involvement (e.g., PIN and AIN) [25,26].
(d) Classic NA is historically associated with a spontaneous and quite good prognosis, while hourglass constriction/nerve torsion is usually associated with a bad prognosis, unless neurosurgical treatment is undergone [8,27].

Moreover, the generic evidence of a common role of nerve inflammation is not decisive proof. It would be hard to say, for example, that multifocal motor neuropathy and chronic immune-mediated demyelinating polyneuropathy are the same pathology because they are both autoimmune and immune-mediated diseases. We know that these pathologies are part of the same group of immune-mediated polyneuropathies, including many other different clinical and neurophysiological entities, sharing the involvement of the immune system but with different mechanisms (different autoantibodies, different patterns of cytokines and inflammatory cells). A recent interesting work used neuroimaging to evaluate the nerve aspect in a large series of cases with a clinical diagnosis of NA. In those patients, the authors found four different possible aspects, i.e., normal nerve, nerve hypertrophy, hourglass constriction and nerve torsion, meaning that acute mononeuropathies with clinically similar characteristics can be associated with very different nerve appearances [28]. A more recent work demonstrated that an hourglass constriction might be already present 12 h from the clinical onset of symptoms and signs of acute mononeuropathy [29]. In our opinion, this finding contradicts the model of hourglass constriction as a progression from nerve swelling in NA. If true, we hypothesize that the first phase of swelling is almost or completely asymptomatic. On the other hand, we know that the correlation between nerve swelling and symptoms is well established; i.e., it is well documented that in NA, the main anatomical feature in many cases is nerve swelling.

Thus, we propose the nomenclature of “acute not traumatic mononeuropathies/plexopathies” for an acute nerve palsy caused by different etiologies for vasculitis and inflammatory/genetic reasons, including typical NA and hourglass constriction/nerve torsion, each maintaining their own specificity.

## 3. What Is the Best Diagnostic Approach for These Conditions?

### 3.1. Neuroimaging

**Figure 2:**
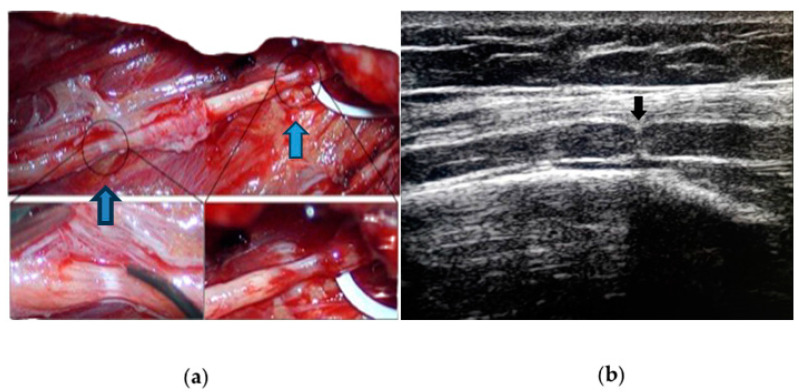
(**a**) Intraoperative appearance of spontaneous (blue arrows) double left radial nerve constriction; (**b**) preoperative ultrasound of the same nerve (black arrow) showing a typical hourglass constriction, with the indentation of the hyperechoic epifascicular epineurium.

It is now well accepted that nerve imaging is necessary for identifying hourglass constrictions/nerve torsion pre-surgically in patients with acute mononeuropathy/plexopathy. Ultrasound (US) is a very reliable and cost-effective technique which carries a high rate of identification and localization of the constriction [30]. It is a useful tool for diagnosis, and it is consistent with intraoperative findings, as shown in Figure 2.

In hourglass constriction when following the nerve course with a transverse scan, there is a sudden and usually marked reduction in the nerve cross-sectional area, usually accompanied by the hyperechoic aspect of the nerve with loss of fascicular structure. Proximally and distally to this site, the cross-sectional area of the nerve is usually increased. At this level, the fascicles are usually increased in size with an aspect varying from hypo- to hyperechoic, probably according to the prevalence of oedema and fibrosis.

Longitudinal scans show the typical appearance of an hourglass-like lesion [31,32]. Nevertheless, US may fail to identify the constriction if the involved nerve is too deep or located under the bones or other body structures blocking the ultrasound beam. Moreover, the assessment is highly operator-dependent. MRI, particularly 1.5 and 3 Tesla scanning, has an important role [33], although it is more expensive, more time-consuming and not widely available in clinical practice. As in the case of US, MRI is highly operator-dependent, especially when it comes to the choice of the appropriate protocol (this can also be quite time-consuming) and imaging interpretation. It can immediately show, proximal to the constriction site, peripheral signal hyperintensity and central hypointensity on intermediate-weighted FSE and/or fat-suppressed imaging, orthogonal to the longitudinal axis of the nerve (“bullseye sign”) [34]. In general, US affords superior spatial resolution in the near field, and thus, if a constriction is detected, US may be more precise than MRI in measuring constriction severity [25,35,36]. MRI, on the other hand, is likely more sensitive than US in detecting the presence of a constriction given its superior contrast resolution and may show constrictions in deeper nerves not detectable by US [37].

We propose using as the first exam of choice US due to its reliability and cost effectiveness. MRI should be considered as the second option, excluding the cases of nerves that cannot be explored by ultrasound (deep nerves such as the anterior interosseous nerve, lumbar plexus, the more proximal part of the sciatic nerve and some parts of the brachial plexus, such as the portion running under the clavicula). Of course, the skills and know-how of each medical center guide the selection of the best available diagnostic tool.

Neuroimaging techniques can also be very helpful in the differential diagnosis of NA, hourglass constriction and nerve torsion, as in the case of an external mass compressing the nerves (neoplasm, cyst, abscess and every kind of pathological tissue in contact with the nerves), intraneural or perineural neoplastic infiltration and other peripheral nerve inflammatory or infectious conditions. Additionally, dynamic US is helpful for differential diagnosis in patients with entrapment or torsional pathologies, allowing us to evaluate the normal or reduced gliding of the nerve at the pathological site, and in patients with nerve compression by an external mass or in the case of perineural neoplastic infiltration, where the mobility or hypomobility of the nerve are assessed. Further studies on US vascular assessment would be helpful to optimize diagnosis and follow-up after conservative and/or surgical treatments.

However, a gold standard for grading constriction severity is still missing, and this hampers the prognostic role of neuroimaging.

### 3.2. Neurophysiology

Neurophysiology should be performed in all cases and is necessary to confirm the clinical diagnosis in terms of location of the damage and type of nerve damage (demyelination vs. axonal vs. conduction block). Moreover, a broad examination can detect subclinical involvement of more nerves, guiding the diagnosis toward vasculitis or a genetic or inflammatory neuropathy. The correct identification of the pathology and type of damage is of the uttermost importance for the prognosis and guiding treatment as well. The neurophysiological correlate of hourglass constriction is still not so clear and in many works is not well specified. This is probably related to the absence of a focus on this aspect in many works that are not reported by neurologists or neurophysiologists. Our clinical experience suggests that the typical damage associated with hourglass constriction/nerve torsion is severe axonal loss [26]. This is in line with the usually reported long latency between the diagnosis and the clinical recovery, when it occurs. In our opinion, the presence of a conduction block should push examiners toward a different diagnosis. It is important to consider some limitations of neurophysiological examination related to the timing of the exam with respect to the onset of symptoms. To avoid a misinterpretation of neurophysiological data, nerve conduction studies should be performed at least ten days after the clinical onset. This is in fact the time required for complete Wallerian degeneration of axons after acute damage [29]. An examination carried out too early can underestimate the entity or the type of damage, leading, for example, to a misdiagnosis of conduction block in the case of axonal damage. This can occur because the stimulation of the nerve proximal to the site of damage does not evoke any potentials (or potentials of reduced amplitude), while stimulation distal to the damage is able to activate the distal portion of the axons, which do not degenerate during the time window of almost 10 days needed to complete the Wallerian degeneration. In the case of the absence of a clear hourglass constriction or nerve torsion, differential diagnosis should be investigated [26].

### 3.3. Differential Diagnosis of Acute Mononeuropathies and Related Examinations

Acute mononeuropathies are usually caused by compression (increased risk in cases of diabetes, hypothyroidism, weight loss or gain, alcoholism, acromegaly); all these pathologies should be considered. The evidence of neurophysiological and neuroimaging signs of nerve sufferance in a typical site of entrapment should suggest this diagnosis. There are, however, other pathologies that can cause isolated mononeuropathy, e.g., in some cases, the early manifestation of multineuropathy. Among these, there are systemic and non-systemic vasculitis, infectious diseases (leprosy, HIV), inflammatory diseases (sarcoidosis, MMN, CIDP variants) and toxic, metabolic (diabetes) and hereditary diseases (HNPP, hereditary neuralgic amyotrophy).

Peripheral nervous system impairment is frequent in most necrotizing vasculitis, with an ischemic–hypoxic mechanism due to arterial vessel occlusion secondary to vessel wall necrosis, mainly causing multineuropathies. This can occur both in primary forms (such as polyarteritis nodosa, Churg–Strauss syndrome, Wegener’s granulomatosis) and secondary forms (in the course of neoplasms, infectious diseases such as HCV, connective tissue diseases such as SLE, rheumatoid arthritis, Sjogren’s syndrome, scleroderma, rheumatic fever, amphetamines use) [38,39].

Infectious disease can cause direct nerve involvement, as in the case of leprosy [40]. Leprosy neuropathy is the second most common neuropathy in the world (after diabetic neuropathy), and it is due to direct invasion of the skin and nerves by *M. leprae*. Leprosy is endemic in tropical and subtropical regions; in other areas, it is suspected in people from or who have stayed in endemic areas. The diagnosis is confirmed by histological examination of the cutaneous lesions with evidence of acid-resistant bacteria.

Opportunistic CMV infection in HIV causes lumbosacral polyneuropathy but can also cause predominantly sensitive multineuropathy with pain and dysesthesia. Lyme disease can manifest as multineuropathy, mononeuropathy or brachial plexopathy, which can mimic brachial neuritis [41].

Chronic hepatitis from HCV (and HBV) carries a risk of developing predominantly axonal sensory and sensorimotor poly- and multineuropathies usually associated with cryoglobulinemia (the presence of serum immunoglobulins which precipitate at low temperatures and solubilize at 37°) or autoimmune vasculitis [42].

Other rare causes are possible. Sarcoidosis is a chronic multisystem inflammatory granulomatosis, which in 5–10% of cases can affect the nervous system and in 1% the peripheral nervous system [43,44]. Radiculopathies or mono-multineuropathies due to granulomatous infiltration, usually slowly progressive, may occur.

Multifocal motor neuropathy (MMN) may present initially as mononeuropathy and is neurophysiologically characterized by the presence of conduction blocks [45,46].

In multiple myeloma, there may rarely be multineuropathy caused either by infiltration of malignant plasma cells or by interstitial deposition of paraprotein or light chains [47,48].

Diabetes is the most frequent cause of neuropathy, and the main risk factors are the duration of the disease and the blood sugar level [49]. It can cause different clinical pictures. While the typical and more common symmetrical polyneuropathy has a predominantly toxic–metabolic etiology, the rarer mono-multineuropathic forms are due to vascular alterations in the vasa nervorum. The cranial nerves can be affected, particularly the third, but peripheral nerves of the limbs and trunk can also be impacted; in both cases, the genesis is ischemic. A typical form is Garland’s diabetic amyotrophy, which begins with intense pain in the muscles of the pelvis and thigh and is followed by rapidly worsening hypotrophy that can resolve over months or years. It is independent of the duration of the disease, and the most accredited hypothesis is that it is an acute ischemic radicle/plexopathy.

Environmental and occupational exposure to lead, which is still widespread in developing countries, can cause axonal motor neuropathy, mainly of the radial nerve (with hand drop) but also of the tibialis anterior (foot drop).

Acute intermittent porphyria is the most common of the hepatic porphyrias. It is caused by a deficiency of the porphobilinogen synthetase enzyme and manifests as attacks of abdominal pain associated with fever, vomiting and leukocytosis. Sometimes there is neurological involvement with acute onset motor deficits and a multineuropathic distribution [50].

Hereditary neuropathy with liability to pressure palsies (HNPP), a rare autosomal dominant hereditary neuropathy caused by deletion of the *PMP22* gene, is a polyneuropathy conferring an increased susceptibility to acute damage from the compression or entrapment of peripheral nerves. It manifests usually as acute transient peripheral paralysis with a mononeuropathic distribution that is not usually accompanied by strong pain. The nerve damage can regress spontaneously within a few days or weeks or persist for a long time. From a neurophysiologic point of view, the typical damage is neuroapraxia in a usual site of entrapment. Some axonal damage usually co-occurs, especially in cases with a prolonged clinical deficit [51,52].

NA can sometimes be hereditary (hereditary neuralgic amyotrophy). This autosomal dominant hereditary form of neuralgic amyotrophy is caused by mutations in the *SEPT9* gene, leading to recurrent episodes of typical brachial neuritis [53,54].

In order to make a correct diagnosis, a full anamnesis could be very helpful. Diabetes, a history of rheumatic diseases, staying in or coming from areas at risk for infectious pathologies and exposure to industrial toxicants could indicate diagnosis. Diagnostic tests such as glycaemia, creatinine, uremia, blood cell count (eosinophilia), ACE enzyme rate, cryoglobulinemia, cANCA, pANCA, rheumatoid factor, anti-CCP, antiGM1, HCV, HBV, HIV and HCV serology, serum and urine levels of lead, increased erythrocyte protoporphyrin and urinary excretion of delta-aminolevulinic acid and coproporphyrin should be performed and can help in establishing the correct diagnosis. Other exams, such as genetic tests for HNPP, skin–mucosal biopsy or, in selected cases, biopsy of an affected nerve, can sometimes contribute important information.

## 4. What Is the Best Treatment Approach for These Conditions?

According to a Cochrane review, there are limited conservative treatment options in NA, and there is no evidence from randomized trials to support any particular form of treatment. In particular, some evidence suggests that early corticosteroid therapy may be effective against pain and lead to an earlier recovery, but there is no evidence of significant long-term improvement if compared with patients not treated with corticosteroids [55].

There is no consensus on hourglass constriction treatment either. Conservative options include observation, resting and physical therapy to cope with muscle weakness, along with medications to control pain, nonsteroidal anti-inflammatory drugs and steroids, but there is no evidence of the effectiveness of these approaches. Some authors have suggested treating patients conservatively for 3 to 6 months, and afterward considering surgery if no improvements occur [56,57]. However, in a recent study, microsurgical epineurolysis and perineurolysis were associated with significantly improved clinical and nerve regeneration at an average follow-up of 14.8 months compared with nonsurgical management in patients with a previous clinical diagnosis of NA [58].

The type of surgical treatment implemented also varies in the literature. The most adopted is internal neurolysis, which allows a decompression of the internal components of the nerve promoting recovery [59].

Resection of the affected segment with direct neurorrhaphy, or with the interposition of an autologous nerve graft, has also been reported. The use of more complex surgical techniques, such as tendon transfers, is sporadic [60]. This technique should be limited to cases without any chance of spontaneous recovery, such as after failed neurosurgical treatment, or when severe chronic muscle atrophy and fibrotic substitution are observed (meaning at least after more than 1 year after the onset) [61]. In this case, neurophysiology can be useful in finding surviving muscle fibers (even if denervated) since the complete absence of fibrillation potentials indicates the absence of muscle fibers available for reinnervation. In other words, muscle fibers that are still alive generate fibrillation potentials, and so their absence, indicating the complete or sub-complete degeneration of muscle fibers, hampers any surgical attempt at reinnervation.

The prognosis is generally favorable after surgery, with a high rate of good motor recovery.

Surprisingly, overall, the surgical technique seemed to not influence the clinical outcome. This finding might simply signify that the chosen surgical treatment was appropriate for the nerve lesion, but still, the literature lacks the elements necessary to correlate the grade of nerve lesion to the best possible treatment, and both to the outcome. More data regarding the correlation between a common severity grading and the postoperative outcome are needed, as well as further parameters to predict the clinical outcome. The parameters that should be considered for this purpose could be the entity of constriction, the presence on the same nerve of more sites of constriction (as described in some cases) and the severity of nerve damage, assessed through neurophysiology. It was suggested, in previous studies, that constriction lower than 75% of the nerve diameters is more likely to improve spontaneously, with some authors proposing in these cases a strategy of wait and see [6]. However, there is no consensus on this. Moreover, it is reasonable to hypothesize worsened spontaneous improvement in the case of more sites of nerve constriction. From a neurophysiological point of view, the presence of complete axonal loss could be a parameter indicating rapid neurosurgical treatment (on the contrary, the partial spreading of a nerve could suggest a conservative approach). Supportive treatment could be useful especially in cases with strong pain. In these cases, drugs for neuropathic pain should be preferred. There is no consensus, due to the lack of data, about the use of corticosteroids that can be used in the acute phase.

In compressive neuropathies, prolonged compression may lead to demyelination and axonal loss, as well as nerve fascicle swelling, leading to epineural fibrosis. In these cases, which share similarities with cases of hourglass constriction, ultrasound-guided hydrodissection with different injectates (for example, normal saline, corticosteroids, local anesthetics, dextrose and platelet-rich plasma) has been demonstrated to provide not only a mechanical effect of releasing and decompressing the entrapped nerves but also a pharmacological effect relieving pain and promoting nerve healing through numerous mechanisms [25,37]. At the moment, there are no studies on the use of ultrasound-guided hydrodissection in the treatment of hourglass constriction, but it is a hypothetically minimally invasive option that should be further studied.

Since hourglass constriction and nerve torsion have a different treatment and prognosis than other cases of acute mononeuropathy/plexopathy without evidence of nerve constriction or torsion, it is of the uttermost importance to perform a correct diagnosis.

**Scheme 1:**
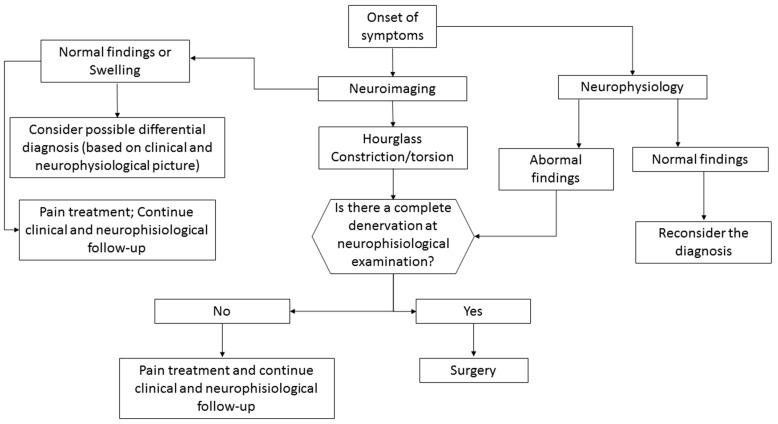
Flow chart for the management of acute non-traumatic mononeuropathies/plexopathies.

For this purpose, we propose a flow chart (revised from Gstoettner et al. [6]) for the management of acute non-traumatic mononeuropathies/plexopathies, mainly focused on hourglass constriction, shown in Scheme 1. The flow chart is based on the complementary role of neurophysiology and neuroimaging, considering their points of strength and limitations. Although neurophysiology is important in the diagnosis of mononeuropaties/plexopathis to confirm the peripheral nerve involvement in terms of location and type of nerve damage, it still has some limitations, mainly related to the timing and the difficulty in exploring some nerves due to their anatomical location. Moreover, the same kind of damage can be present in different pathologies, and hourglass constriction and nerve torsion diagnoses cannot be made with neurophysiology (only hypothesized in the framework of a particular clinical contest).

On the other hand, neuroimaging is needed in order to diagnose hourglass constriction and nerve torsion before surgery but cannot identify the type of damage or quantify it.

For these reasons, we propose using neuroimaging and neurophysiology in parallel and interpreting the combination of information provided by the different techniques in the framework of the clinical contest.

## Figures and Tables

**Table 1:** Main differences between NA, hourglass constriction and nerve torsion, related to the type of nerve involved, clinical features and neurophysiological features.

	Neuralgic Amyotrophy	Hourglass Constriction/Nerve Torsion
**Site**	Mainly brachial plexus	Radial, anterior and posterior interosseous nerves
**Clinical picture**	Strong and invalidating pain (lasting usually more than 24 h), exclusive or main motor fiber involvement also in the case of mixed nerves	Pain that is not invalidating and of a short duration (lasting usually less than 24 h)
**Neurophysiology**	Exclusive or main motor fibers involvement also in the case of mixed nerve involvement	Same involvement of motor and sensory nerves in the case of mixed nerve involvement

## Data Availability

No new data were created or analyzed in this study. Data sharing is not applicable to this article.
